# Comparative efficacy of different acute reperfusion therapies for acute ischemic stroke: a comprehensive benefit–risk analysis of clinical trials

**DOI:** 10.1002/brb3.279

**Published:** 2014-09-11

**Authors:** Georgios Tsivgoulis, John Alleman, Aristeidis H Katsanos, Andrew D Barreto, Martin Kohrmann, Peter D Schellinger, Carlos A Molina, Andrei V Alexandrov

**Affiliations:** 1 Department of Neurology, University of Tennessee Health Science CenterMemphis, Tennessee; 2 Second Department of Neurology, “Attikon Hospital”, School of Medicine, University of AthensAthens, Greece; 3 International Clinical Research Center, St. Anne's University Hospital in BrnoBrno, Czech Republic; 4 Cerevast Therapeutics, Inc.Redmond, Washington; 5 Department of Neurology, University of IoanninaIoannina, Greece; 6 Department of Neurology, University of Texas-Houston Medical SchoolHouston, Texas; 7 Department of Neurology, University Clinic at ErlangenErlangen, Germany; 8 Departments of Neurology and Neurogeriatry, Johannes Wesling Clinic MindenMinden, Germany; 9 Neurovascular Unit, Department of Neurology, Hospital Vall d'HebronBarcelona, Spain

**Keywords:** Acute stroke, analysis, benefit-to-risk ratio, reperfusion therapies

## Abstract

**Background:**

Numerous acute reperfusion therapies (RPT) are currently investigated as potential new therapeutic targets in acute ischemic stroke (AIS). We conducted a comprehensive benefit–risk analysis of available clinical studies assessing different acute RPT, and investigated the utility of each intervention in comparison to standard intravenous thrombolysis (IVT) and in relation to the onset-to-treatment time (OTT).

**Methods:**

A comprehensive literature search was conducted to identify all available published, peer-reviewed clinical studies that evaluated the efficacy of different RPT in AIS. Benefit-to-risk ratio (BRR), adjusted for baseline stroke severity, was estimated as the percentage of patients achieving favorable functional outcome (BRR1, mRS score: 0–1) or functional independence (BRR2, mRS score: 0–2) at 3 months divided by the percentage of patients who died during the same period.

**Results:**

A total of 18 randomized (*n* = 13) and nonrandomized (*n* = 5) clinical studies fulfilled our inclusion criteria. IV therapy with tenecteplase (TNK) was found to have the highest BRRs (BRR1 = 5.76 and BRR2 = 6.82 for low-dose TNK; BRR1 = 5.80 and BRR2 = 6.87 for high-dose TNK), followed by sonothrombolysis (BRR1 = 2.75 and BRR2 = 3.38), while endovascular thrombectomy with MERCI retriever was found to have the lowest BRRs (BRR1 range, 0.31–0.65; BRR2 range, 0.52–1.18). A second degree negative polynomial correlation was detected between favorable functional outcome and OTT (*R*^2^ value: 0.6419; *P* < 0.00001) indicating the time dependency of clinical efficacy of all reperfusion therapies.

**Conclusion:**

Intravenous thrombolysis (IVT) with TNK and sonothrombolysis have the higher BRR among investigational reperfusion therapies. The combination of sonothrombolysis with IV administration of TNK appears a potentially promising therapeutic option deserving further investigation.

## Introduction

Intravenous thrombolysis (IVT) with recombinant tissue plasminogen activator (tPA) remains the only approved therapy for acute ischemic stroke that can reverse neurological deficits and improve functional outcome (Furie et al. 2011). However, numerous additional acute reperfusion therapies (RPT) are being currently investigated for the treatment of acute ischemic stroke (AIS). These RPT include intra-arterial thrombolysis, IV thrombolysis with tenecteplase (TNK), sonothrombolysis, and mechanical thrombectomy (Hennerici et al. 2013). The main obstacle to a more widespread administration of the aforementioned RPTs is that there are limited randomized or observational data directly comparing these interventions (Barreto and Alexandrov 2012).

Benefit–risk analysis evaluates whether a radical procedure (medical or surgical) is worth the risk to the patient, taking into account the potential benefits of a successful outcome because of this particular procedure (Edwards et al. 1996). Moreover, benefit–risk analysis—using both qualitative and quantitative assessments—allows for indirect comparisons between different therapies, which are evaluated for the same indication (Guo et al. 2010).

In view of the former considerations we sought to conduct a comprehensive benefit–risk analysis of the available clinical randomized and nonrandomized studies that assessed different acute RPT, and to investigate the clinical efficacy of each intervention in relation to the onset-to-treatment time (OTT).

## Methods

We conducted a comprehensive literature search through the MEDLINE, (EMBASE, and the CENTRAL Register of Controlled Trials) and the Internet Stroke Center databases to identify all available clinical studies that assessed different RPT in acute ischemic stroke. Our search strategy was based on the combination of terms: “stroke,” “cerebral ischemia,” “thrombolysis,” “recombinant tissue plasminogen activator,” “alteplase,” “tenecteplase,” “thrombectomy,” “sonolysis,” and “sonothrombolysis.” Last literature search was conducted on February 21st, 2014.

Studies were included in the qualitative analysis if they presented published and peer-reviewed results from Phase 2 or later, randomized or multicenter controlled trials. Reference lists of all articles that met the inclusion criteria and references of relevant review articles were examined to identify studies that may have been missed by the initial database search. References of retrieved articles were also screened. Duplicate publications were excluded from further evaluation.

We evaluated data and conducted a benefit–risk analysis from the studies that provided the following data for each group (target, control, or cohort) within the study:

1. Admission or baseline stroke severity quantified by the National Institutes of Health Stroke Scale (NIHSSb),
2. Time elapsed from OTT
3. Incidence of symptomatic intracranial hemorrhage (sICH)
4. Incidence of mortality/death
5. 90-days posttreatment modified Rankin Scale scores (90d mRS)

A favorable functional outcome (FFO) was defined as a mRS score of 0–1 at 3 months following stroke onset. Functional independence (FI) was documented as a mRS score of 0–2 at 3 months. Dependency was categorized as a mRS score of 3–5, and death was defined as a mRS score of 6.

### Statistical analyses

The benefit-to-risk ratios (BRRs), adjusted for baseline stroke severity, were then estimated separately for all different arms of each study using the following formulas: 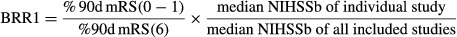


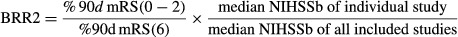


The numerator in the first term of the formulas above expresses the measure of benefit in which the patient has FFO (mRS = 0–1) or FI (mRS = 0–2) at 90 days after stroke onset, while the denominator indicates the percentage of deceased patients during the same study period. As baseline stroke severity is a known predictor of 3-month outcome (Muir et al. 1996), we added the second ratio in the formula (median NIHSSb of individual study divided by median NIHSSb of all included studies) to provide a simple linear method of compensating for the NIHSSb reported in each study. If the ratio is less than 1, it indicates a patient sample which had lower severity strokes at the beginning of therapy and acts to lower the BRR for those interventions with less severe stroke, while improving the BRR for those studies with more severe baseline strokes.

No adjustment for the OTT in the BRR formulas was added, since our prespecified hypothesis was to investigate the association of FFO and BRR with OTT. Subsequently, the correlation of OTT with the 90-days outcome was evaluated using the appropriate correlation coefficients and regression models (linear or polynomial). The Statistical Package for Social Science version 13.0 for Windows (SPSS Inc., Chicago, IL) was used for statistical analyses.

## Results

A total of 18 separate randomized (13 studies) or observational (5 studies) clinical trials fulfilled our prespecified inclusion criteria. These studies were evaluating IVT with tPA (tissue plasminogen activator for acute ischemic stroke; The National Institute of Neurological Disorders and Stroke rt-PA Stroke Study Group 1995) or TNK (Parsons et al. 2012), sonothrombolysis in CLOTBUST (Alexandrov et al. 2004), intra-arterial thrombolysis in PROACT I (del Zoppo et al. 1998), PROACT II (Furlan et al. 1999), IMS III (Broderick et al. 2013), SYNTHESIS pilot (Ciccone et al. 2010), and expansion study (Ciccone et al. 2013) and acute thrombectomy/thromboaspiration with different retrievers: MERCI (Smith et al. 2005), multi-MERCI (Smith et al. 2008), PENUBRA Pivotal Stroke Trial (2009), and Post Market Experience (Tarr et al. 2010), SWIFT (Saver et al. 2012), TREVO (San Roman et al. 2012), TREVO 2 (Nogueira et al. 2012), and MR Rescue (Kidwell et al. 2013). The baseline characteristics of the study populations of the included studies are briefly summarized in Table 1.

**Table 1:** Baseline characteristics of included studies.

Study	Category	Intervention	*n*	Age (years)	OTT (h)	Baseline NIHSS	sICH (%)	90d mRS (0–1) (%)	90d mRS (0–2) (%)	90d mRS (6) (%)
CLOTBUST	TCD sonolysis	IV-tPA+TCD	63	67.0 ± 11.9	2.5	17.0	5	42	51	15
NINDS	Placebo/SOC	Placebo	165	66.0 ± 13.0	1.5	15.0	1	26	–	21
PROACT I	Placebo/SOC	Placebo	14	69.6 ± 11.1	5.7	19.0	7	21	–	43
PROACT II	Placebo/SOC	IV-Heparin	59	64.0 ± 14.0	5.1	17.0	2	17	25	27
NINDS	IV thrombolysis	IV-tPA	168	69.0 ± 12.0	1.5	14.0	7	39	–	17
CLOTBUST	IV thrombolysis	IV-tPA	63	70.0 ± 13.1	2.2	16.0	5	29	37	18
IMS III	IV thrombolysis	IV-tPA	222	68.0 (23–84)	2.0	16.0	6	27	40	22
MR rescue	IV thrombolysis	IV-tPA/SOC-Penumbral	34	65.8 ± 16.9	5.8	16.0	6	15	23	9
MR rescue	IV thrombolysis	IV-tPA/SOC-Non-Penumbral	20	69.4 ± 15.9	5.7	20.5	0	6	10	22
SYNTHESIS expansion	IV thrombolysis	IV-tPA	181	67.0 ± 11.0	2.8	13.0	6	35	31	10
SYNTHESIS pilot	IV thrombolysis	IV-tPA	29	64.0 ± 11.7	2.6	16.0	14	28	31	17
TAAIS	IV thrombolysis	IV-TNK-1	25	72.0 ± 6.9	3.1	14.5	4	54	64	8
TAAIS	IV thrombolysis	IV-TNK-2	25	68.0 ± 9.4	3.0	14.6	0	54	64	8
TAAIS	IV thrombolysis	IV-tPA	25	70.0 ± 8.4	2.7	14.0	4	40	44	12
IMS III	Endovascular	IA-tPA+MT	434	69.0 (23–89)	4.2	17.0	6	29	43	20
MERCI	Endovascular	MT-Merci	141	67.0 ± 15.5	4.3	20.1	8		28	44
MR rescue	Endovascular	MT (+IV-tPA)(+IA-tPA)-Penumbral	34	66.4 ± 13.2	6.0	16.0	9	9	14	16
MR rescue	Endovascular	MT (+IV-tPA)(+IA-tPA)-Non-Penumbral	30	61.6 ± 12.0	6.0	20.5	0	6	10	23
Multi-MERCI	Endovascular	MT-Merci	164	68.1 ± 16.0	4.3	19.0	10	–	36	34
Penumbra POST	Endovascular	MT-Penumbra	157	65.0 ± 15.0	4.5	16.0	6	–	41	20
Penumbra PST	Endovascular	MT-Penumbra	125	63.5 ± 13.5	4.3	17.6	11	–	25	33
PROACT I	Endovascular	IA-ProUK	26	66.5 ± 11.0	5.4	17.0	15	31	–	27
PROACT II	Endovascular	IA-ProUK	121	64.0 ± 14.0	4.7	17.0	10	26	40	25
SWIFT	Endovascular	MT-Solitaire	58	67.1 ± 12.0	4.9	17.3	2	26	28	17
SWIFT	Endovascular	MT-MERCI	55	67.1 ± 11.1	5.3	17.5	11	18	27	38
SYNTHESIS expansion	Endovascular	IA-tPA+MT	181	66.0 ± 11.0	3.8	13.0	6	30	42	14
SYNTHESIS pilot	Endovascular	IA-tPA	25	60.6 ± 13.7	3.3	17.0	8	48	56	24
TREVO	Endovascular	MT (+IV-tPA)	60	71.2 ± 12.4	3.5	18.0	12	–	45	28
TREVO 2	Endovascular	MT-Trevo	88	67.4 ± 13.9	4.6	18.3	7	27	40	34
TREVO 2	Endovascular	MT-Merci	90	67.0 ± 14.7	4.5	17.9	9	15	22	24

OTT, onset-to-treatment time; NIHSS, National Institute of Health Stroke Scale; 90d mRS, modified Rankin Scale at 90 days; TCD, transcranial Doppler; IV, intravenous; tPA, alteplase; TNK, tenecteplase; UK, urokinase; SOC, standard of care; IA, intra-arterial; MT, mechanical thrombectomy.

**Figure 1:**
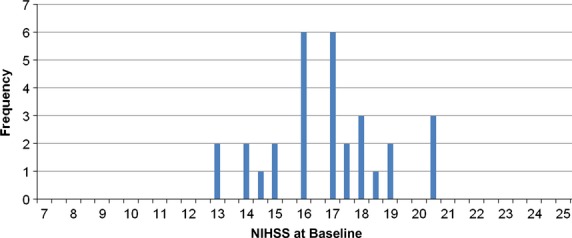
Histogram of baseline NIHSS values across included studies. NIHSS, National Institute of Health Stroke Scale.

In each of the included studies NIHSSb was reported either as a mean and/or a median value. The distribution of the mean/median NIHSSb values that were reported in the studies is shown in Figure1. Because the bulk of the distribution seems to be symmetric with a central tendency of about 17, we choose the value of 17 as the reference NIHSSb value in the BRR formulas to determine the BRR1 and BRR2 in each separate arm of all evaluated studies (Table 2). IVT with TNK yielded the highest BRRs (BRR1 = 5.76 and BRR2 = 6.82 for low-dose TNK-1; BRR1 = 5.80 and BRR2 = 6.87 for high-dose TNK), followed by sonothrombolysis (BRR1 = 2.75 and BRR2 = 3.38). In contrary, endovascular thrombectomy using MERCI retriever appeared to have the lowest BRRs (BRR1 range, 0.31–0.65; BRR2 range, 0.52–1.18).

**Table 2:** Benefit-to-risk analysis of the included studies.

Study	Category	Intervention	BRR1	BRR2
CLOTBUST	TCD sonolysis	IV-tPA+TCD	2.75	3.38
NINDS	Placebo/SOC	Placebo	1.09	–
PROACT I	Placebo/SOC	Placebo	0.56	–
PROACT II	Placebo/SOC	IV-Heparin	0.63	0.93
NINDS	IV thrombolysis	IV-tPA	1.89	–
CLOTBUST	IV thrombolysis	IV-tPA	1.46	1.88
IMS III	IV thrombolysis	IV-tPA	1.14	1.69
MR rescue	IV thrombolysis	IV-tPA/SOC-Penumbral	1.57	2.41
MR rescue	IV thrombolysis	IV-tPA/SOC-Non-Penumbral	0.33	0.55
SYNTHESIS expansion	IV thrombolysis	IV-tPA	2.68	2.39
SYNTHESIS pilot	IV thrombolysis	IV-tPA	1.51	1.69
TAAIS	IV thrombolysis	IV-TNK-1	5.76	6.82
TAAIS	IV thrombolysis	IV-TNK-2	5.80	6.87
TAAIS	IV thrombolysis	IV-tPA	2.75	3.02
IMS III	Endovascular	IA-tPA+MT	1.47	2.14
MERCI	Endovascular	MT-Merci	–	0.75
MR rescue	Endovascular	MT (+IV-tPA)(+IA-tPA)-Penumbral	0.53	0.82
MR rescue	Endovascular	MT (+IV-tPA)(+IA-tPA)-Non-Penumbral	0.31	0.52
Multi-MERCI	Endovascular	MT-Merci	–	1.18
Penumbra POST	Endovascular	MT-Penumbra	–	1.95
Penumbra PST	Endovascular	MT-Penumbra	–	0.79
PROACT I	Endovascular	IA-ProUK	1.14	–
PROACT II	Endovascular	IA-ProUK	1.04	1.60
SWIFT	Endovascular	MT-Solitaire	1.56	1.68
SWIFT	Endovascular	MT-MERCI	0.49	0.73
SYNTHESIS expansion	Endovascular	IA-tPA+MT	1.62	2.24
SYNTHESIS pilot	Endovascular	IA-tPA	2.00	2.33
TREVO	Endovascular	MT (+IV-tPA)	1.70	2.08
TREVO 2	Endovascular	MT-Trevo	0.85	1.26
TREVO 2	Endovascular	MT-Merci	0.65	0.95

BRR, benefit-to-risk ratio; TCD, transcranial Doppler; IV, intravenous; tPA, alteplase; TNK, tenecteplase; UK, urokinase; SOC, standard of care; IA, intra-arterial; MT, mechanical thrombectomy.

**Figure 2:**
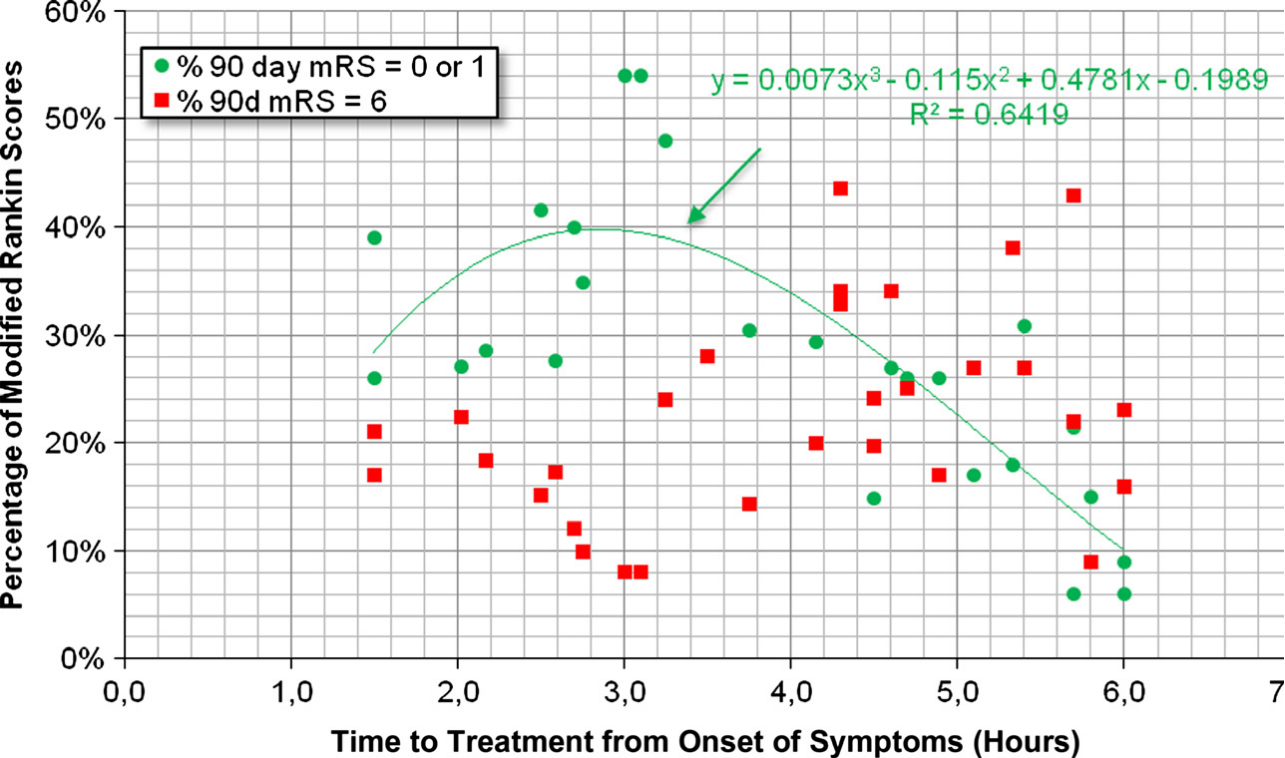
Plot of 90-day modified Rankin Scale scores versus time to treatment.

A second degree negative polynomial correlation (Fig.2) was detected between FFO and OTT (*R*^2^ value: 0.6419; *P* < 0.00001) indicating that approximately two thirds (64%) of the variation in FFO at 3 months across different trials of RPT can be explained by differences in OTT. Interestingly, no association (*P *= 0.527) was detected between mortality and OTT (Fig.2).

**Figure 3:**
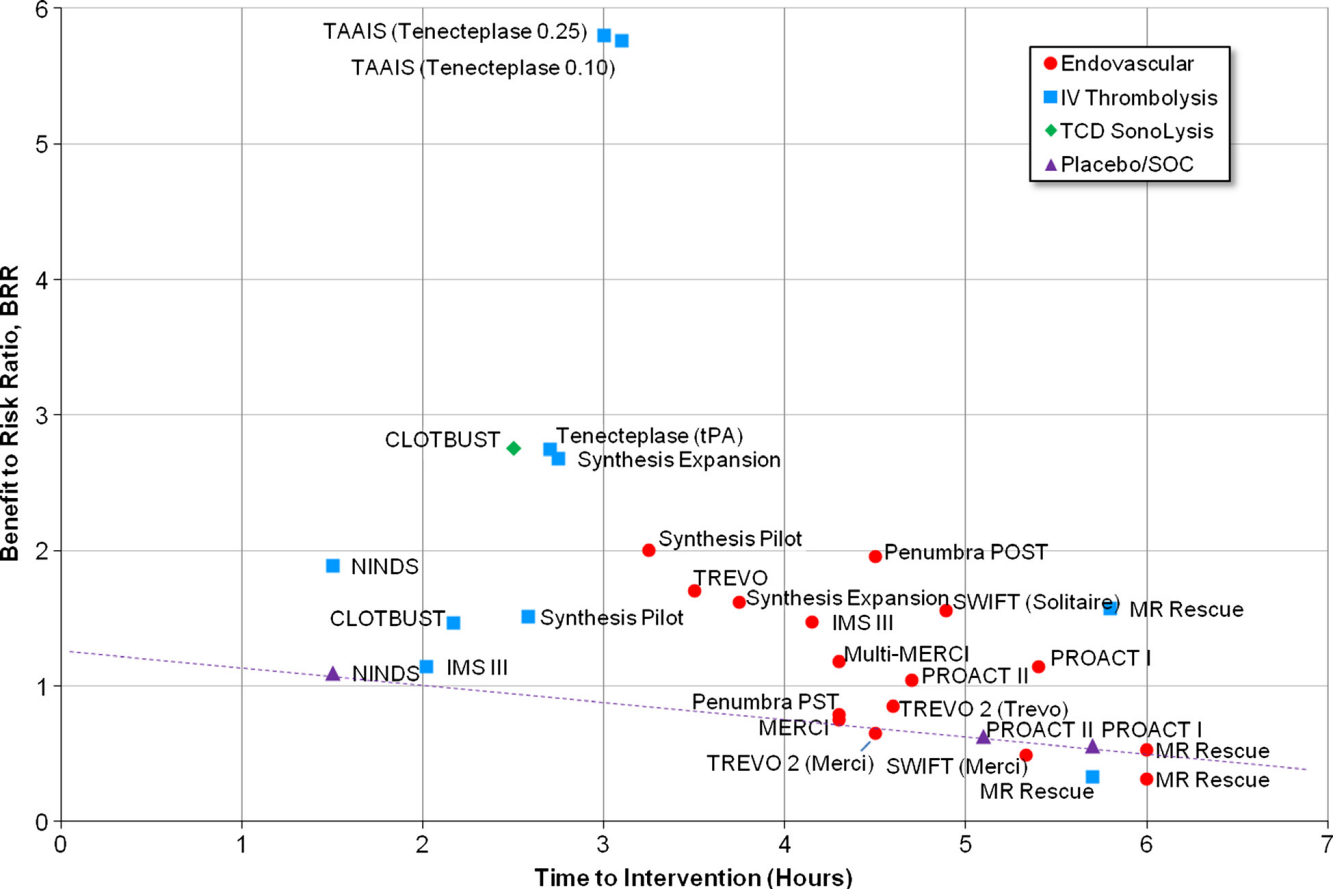
Benefit-to-risk ratio versus onset-to-treatment time. IV, intravenous; TCD, transcranial; SOC, standard of care.

We additionally plotted the BBR values of all trials versus the corresponding OTT (Fig.3), and documented a time dependency of BRR across all different RPT. More specifically, BRR decreased substantially in relation to time in trials evaluating IVT (either with alteplase or TNK), sonothrombolysis, intra-arterial thrombolysis, and mechanical thrombectomy with different retrievers. Moreover, the BRR values of the NINDS placebo intervention (BRR = 1.09) at 1.5 h, followed by the PROACT II placebo (BRR = 0.63) at 5.1 h, and the PROACT I placebo (BRR: 0.56) at 5.7 h lie in a straight line (dashed purple line, Fig.3). This line could presumably be considered as the benchmark trend for standard care without thrombolysis or endovascular intervention, and thus any intervention above this line could be regarded as better than standard noninvasive/nonthrombolytic care and any thrombolytic or endovascular intervention below this line could be considered as having a questionable benefit. Interestingly, the only RPT that exhibited consistently BRR values below this line representing BRR of standard of care was thrombectomy with MERCI device in TREVO 2, SWIFT, and MR RESCUE trials.

## Discussion

We performed a comprehensive benefit-to-risk analysis of the available clinical trials evaluating acute RPT in AIS. IVT with TNK, followed by sonothrombolysis, appear to be associated with the highest BRRs. In contrast mechanical thrombectomy with MERCI device yielded consistently the lowest BRRs across different arms of acute reperfusion trials. Finally, we detected a strong time dependency of BRR in all evaluated reperfusion therapies. This observation further reinforces the concept of “time is brain” in the management of acute cerebral ischemia (Naylor 2007).

The TAAIS (tenecteplase vs. alteplase for acute ischemic stroke) trial (Parsons et al. 2012) shows by far the highest BRR among all studies. This finding may be attributed to the following factors. First, TNK is more effective and safer thrombolytic medication in comparison to alteplase in acute myocardial infarction trials (Barreto and Alexandrov 2012). Additionally, a perfusion lesion 20% or greater than the infarct core on computed tomographic (CT) perfusion imaging at baseline (also referred to as “target mismatch”) and an associated vessel occlusion on CT angiography were mandatory for randomization according to the study's inclusion criteria (Parsons et al. 2012). Consequently these criteria greatly favored the selection of patients most likely to benefit from thrombolytic therapy.

Evidence is still inadequate to conclude for the superiority of a thrombolytic agent, dose, or route of administration in the treatment of acute cerebral ischemia (Wardlaw et al. 2013; Landry et al. 2014). To date, the only approved thrombolytic for the treatment of acute ischemic stroke is t-PA 0.9 mg/kg, while other drugs, doses, or routes of administration are only tested in clinical trial protocols (Wardlaw et al. 2013). The search for other thrombolytic agents has been triggered by the high rates of unsuccessful reperfusion and adverse bleeding episodes observed with tPA (Balami et al. 2013; Rother et al. 2013). TNK is a genetically engineered variant of tPA that has a longer half-life and is more fibrin-specific than t-PA. These properties make TNK a very advantageous thrombolytic to induce faster and more complete clot lysis, with less bleeding complications and early reocclusions (Martinez-Sanchez et al. 2007). An additional benefit is that TNK can be administered by IV bolus infusion, without the need for follow-up infusion (Bivard et al. 2013). Although TNK has already been approved for the treatment of acute myocardial infarction, the use of TNK instead of tPA in the treatment of acute ischemic stroke outside of the setting of a clinical trial still remains unapproved, and should only be tested in clinical trials (Bivard et al. 2013; Behrouz 2014).

Sonothrombolysis is the ultrasound targeting of an arterial occlusive clot in order to accelerate the thrombolytic effect of systemic tPA. Mechanical pressure waves produced by 2 MHz frequency ultrasound energy improve the delivery and penetration of the thrombolytic drug inside the clot (Rubiera and Alexandrov 2010; Alexandrov and Barlinn 2012). The CLOTBUST ultrasound-enhanced thrombolysis (Alexandrov et al. 2004) had one of the highest BRRs despite no target mismatch and lack of NIHSS cutoffs pretreatment. This observation is consistent with a recent meta-analysis indicating that sonothrombolysis with high-frequency ultrasound almost triples the likelihood of complete recanalization and doubles the odds of FFO in comparison to standard IVT (Tsivgoulis et al. 2010). Two other independent meta-analyses have also shown that sonothrombolysis appears to reduce death or dependency at 3 months and to increase recanalization, without further augmenting the risk of symptomatic intracranial hemorrhage (Ricci et al. 2012; Saqqur et al. 2014). At present, sonothrombolysis can be performed at bedside using the available vascular diagnostic transcranial Doppler or transcranial duplex ultrasound systems (Alexandrov 2010). An operator-independent 2-MHz transcranial Doppler device, developed to provide therapeutic ultrasound regardless of sonography skills, is currently being tested in a pivotal multicenter sonothrombolysis efficacy trial (Barlinn and Alexandrov 2013).

It could be hypothesized that if sonolysis was used with other thrombolytics such as TNK, there might be a noticeable increase in BRR. However, it should be noted that not only this approach is to date only hypothetical, but also specific parameters—such as the output power, the duty cycle, the pulse width, the exposure time, and the impact of skull bone characteristics—remain to be defined for the optimal use of this technique (Lapchak et al. 2013).

The low BRRs that we detected consistently across all arms of acute thrombectomy trials using MERCI device indicate that the potential increase in recanalization rates with endovascular reperfusion therapies may not always translate into clinical efficacy. A secondary analysis of IMS III indicated that every 30-min delay in angiographic reperfusion reduced the relative likelihood of a good clinical outcome by 15% in unadjusted analysis and 12% in adjusted analysis (Khatri et al. 2014). Moreover, increased time to reperfusion was associated with a rise in the number of serious adverse events in IMS III (Khatri et al. 2014). Similar results have been reported after analysis of pooled data from MERCI, TREVO, and TREVO 2 trials with an 11% increase in the OR for functional dependence for every 30 min delay from symptom onset to endovascular treatment time (Shi et al. 2014). Consequently, the delayed OTT in combination with the longer symptom onset to reperfusion time documented in trials evaluating the safety and efficacy of mechanical thrombectomy with MERCI device (Smith et al. 2005, 2008) may account for the poor clinical efficacy of MERCI in the present benefit–risk analysis. An alternative plausible explanation may be related to the potential superiority of stent retrievers in achieving fast recanalization in comparison to MERCI retriever.

Certain limitations of this report need to be acknowledged. First, in the present benefit-to-risk analysis we made an indirect comparison of treatment effects using the BRR values. BRR provides a simple quantification method that could lead to wrong conclusions, if the underlying assumptions and limitations are not well understood. In many benefit–risk analyses neither the benefits nor the risks are appropriately compared quantitatively, and thus the final assessment is largely qualitative and somewhat subjective. In the present analysis, we included and compared both qualitatively and quantitatively data from randomized and nonrandomized clinical studies with substantial differences in baseline characteristics. Additionally, no control or multivariate adjustment for potential confounders – except for NIHSSb and OTT – was made. For example, the standard or care for the acute ischemic stroke has been improving over the years, but this variable is impossible to compare among trials.

In conclusion, a strong time dependency of BRR was found among acute reperfusion trials underlying the urgency for therapy initiation as soon as possible in AIS. IVT with TNK appears to be associated with the highest BRR, followed by sonothrombolysis. The combination of sonolysis with IV administration of TNK could be a promising and attractive novel therapeutic option that deserves further investigation. However, each of the aforementioned therapeutic options (sonolysis and TNK) should be first tested and proven to be superior (or at least equal) against rT-PA before investigating the combination of these modalities against the current standard of care.
